# Disempowered Mothers Have Undernourished Children: How Strong Is the Intrinsic Agency?

**DOI:** 10.3389/fpubh.2022.817717

**Published:** 2022-02-03

**Authors:** Sujan Poudel, Chiranjivi Adhikari, Rajesh Kumar Yadav, Dipendra Kumar Yadav, Deependra Kaji Thapa, Mihajlo Jakovljevic

**Affiliations:** ^1^School of Health and Allied Sciences, Pokhara University, Pokhara, Nepal; ^2^Indian Institute of Public Health Gandhinagar (IIPHG), Gandhinagar, India; ^3^Birat Nepal Medical Trust (BNMT), Kathmandu, Nepal; ^4^Nepal Public Health Research and Development Center (PHRD Nepal), Kathmandu, Nepal; ^5^School of Nursing, Midwifery and Social Sciences, CQUniversity, Rockhampton, QLD, Australia; ^6^Institute of Comparative Economic Studies, Hosei University Faculty of Economics, Tokyo, Japan; ^7^Department Global Health Economics and Policy, University of Kragujevac, Kragujevac, Serbia

**Keywords:** autonomy, children, empowerment, rural, undernutrition, women, intrinsic agency

## Abstract

**Objective:**

Undernutrition is one of the leading causes of morbidity and mortality among under-five children, particularly in low-and-middle-income countries. Since women, including mothers, are the primary caregivers of their children, their empowerment status can inherently influence children's nutritional status. Empowerment is, mainly, an intrinsic agency developed as an affective domain trajectory or attitude that guides the skill or behavior. This study aimed to assess the association between women's empowerment and nutritional status of their children.

**Methods:**

A cross-sectional study was carried out among 300 mothers having children aged 6–59 month in rural municipalities of Kaski district in Nepal. Face to face interview and various anthropometric measurements were used to collect data. Chi-square test was performed to assess the association between women's empowerment and children's nutritional status, and multivariable logistic regression was used to assess the strength of association.

**Results:**

Of 300 mothers having 6–59 months' children, nearly half (49%) were highly empowered while around 38% children were in poor nutritional status. More than one-fourth (26.7%) children were stunted, 7% were wasted, 17.7% were underweight, and in overall, nearly 38% were in poor nutrition. There was a five-fold increase in odds of wasting, thirty-fold increase in odds of stunting, and twenty-nine-fold increase in odds of underweight among children whose mothers had low empowerment status compared to their counterparts.

**Conclusion:**

Overall, this study exhibited that maternal empowerment strongly affected children's nutritional status, especially stunting and underweight. Thus, intrinsic factor, mainly education and community membership are suggested to empower them for making their own decisions. Interventions aiming to improve nutritional status of children should include women empowerment incorporating dimensions of material resources. Further empirical evidence is required from trials and cohort studies.

## Introduction

Health, physical growth and development, academic performance, and progress in life are determined by the nutritional status of children (1). Undernutrition is one of the leading causes of morbidity and mortality among under-five children in low-and-middle-income countries (LMICs) (2), and estimated to prevail among one-third of children worldwide (3). The lack of appropriate care for both women and children is one of the underlying factors of undernutrition among children (4). These underlying trends have been largely affected by health expenditures in low and middle income countries (LMICs) in recent decades (5). Development assistance for health (DAH) provided to the largest multilateral agencies by donors among wealthiest OECD countries appears to be shrinking (6), yet national spending on health and social insurance including early childhood support continues to grow across LMICs (7). Leaders in such a growth across the Global South remain the emerging markets such as the Brazil, Russia, India, China, and South Africa (BRICS) (8) and the countries of emerging seven markets (EM7) (9).

Although the global trend of child nutritional status in LMICs has improved in the last few decades, still 28% of under-five children are stunted. One of the core causes for child undernutrition is the lack of appropriate care for women and children (4, 10). It is extensively acknowledged that women play a vigorous role in children's nurture and also for enhancing child health capabilities throughout the childhood period (11). Enhancing women's status both at domestic and public spheres is important to enhance child wellbeing including the empowerment of nutritional status. Scholars define women empowerment as women's sense of self-worth; their right to have and determine choices; access to opportunities and resources; and power to control their own lives, both within and outside their home (12). Women empowerment is a multidimensional attribute having some 200 indicators (13) roughly rounding off under three latent group of variables- the assets [information, household (HH) material resources, and house/land ownership], the instrumental agency (financial autonomy/purchasing decision, decisions in healthcare, and family planning), and intrinsic agency (group membership, education, spousal communication, and attitude toward intimate partner violence) (14–17). Basically, the third one, intrinsic agency, is more inherent, having sense of self-worth, developed as an attitudinal belief that guides certain skills and behaviors and also orthogonal and deterministic to the instrumental agency like decision makings (18, 19).

By 2030, ending preventable deaths of newborns and under-five children, ensuring women's full and effective participation and equal opportunities for leadership at all levels of decision making in political, economic, and public life in all countries were the established target of Goal 3 and Goal 5 of the sustainable development goals (SDG) (20). In this scenario, this study aimed to assess the relationship between women's empowerment and nutritional status of their children.

## Materials and Methods

### Study Design

A cross-sectional analytical study was conducted in four rural municipalities of Kaski district in Nepal. The study participants were mothers having children aged 6–59 months.

### Sample Size Calculation and Technique

The sample size was determined as 300 by using the formula for cross-sectional studies taking the prevalence (p) of stunting as 28.9% (that yielded higher sample size than wasting (5.8%) and underweight (14.9%) (21) with a 95% confidence interval, allowable error of 5%, and applying a finite population correction.

From a list of 6,023 eligible children in rural municipalities, sample size for each of the 25 clusters have been calculated by probability proportional to size (PPS) technique. In order to select the first sample in each cluster, we used WHO EPI method (22).

### Measures

Face to face interview and anthropometric measurements were used as data collection techniques. Women's empowerment was assessed using a women empowerment index (WEI) which includes five indicators, i.e., women's involvement in household decision-making, women's membership in community groups, women's cash earning, women's ownership of house/land and women's education (14). Tools were translated in the Nepali language, translation validity maintained with iterative process, and used for the interviews. Economic status was calculated based on the international wealth index (IWI) (23). Anthropometric measurements of the children were assessed by the researcher as per the WHO guidelines (24). Age of the child was determined by asking with respondent and further cross-checked with birth certificate or immunization card. Children up to 24 months and not able to stand by their own were weighed with the analog Salter type baby weighing scale with nearest 0.01 Kg and those aged 25–59 months were weighed using bathroom scale with the nearest 0.5 Kg (25). In order to check the validity of salter scale and weighing scale, weights that were proven valid by Nepal Bureau of Standards and Metrology were used. After measuring the weight of every five children scales were rechecked by using standard weights of 5 kg and manually adjusted. Height of the child was measured to the nearest 0.1 cm and to validate, we repeated the measurement twice to obtain two readings within 0.2 cm and the average of two closest measurements were recorded (25).

### Study Variables and Statistical Analyses

We included socio-demographic variables as background variables in the study and dummied as confounders. Sociodemographic variables included age of mother and child, religion, ethnicity, type of family, number of U5 children, the women had, household economic status and the sex of child. The independent variable was women's empowerment and included five indicators- decision-making; involvement in community groups; independent cash earing; house/land ownership; and educational status. Empowerment level was categorized as low, moderate, and high. The dependent variable of the study was the nutritional status and assessed as stunting, wasting, and underweight. Socio-demographic variables, mother's empowerment, and nutritional status of 6–59 months children were described per protocol. In the first analysis, the mother's empowerment status was associated to children's nutritional status using chi-square and unadjusted odds ratio. For the adjustment, then, we filtered out and selected only the significant (*p* < 0.05) two socio-demographic variables-maternal age (*p* = 0.025), and number of children (*p* = 0.003); from the seven variables included in primary analysis. We also checked autocorrelation of these two variables with maternal empowerment where the highest correlation, among all, was observed as *r* = 0.18 (*p* = 0.002) between the number of children and maternal empowerment score. Since it was low (*r* = 0.18), so, we proceeded for the second step for adjustment and calculated the adjusted odds ratios.

### Ethical Consideration

Ethical approval was obtained from the Institutional Review Committee (IRC), Pokhara University. Written informed consent were taken from selected rural municipalities for conduction of study in those areas. Participants were not subjected to any sort of harm and they were informed about their autonomy to withdraw from the study at any time during the study. Confidentiality of their information was fully maintained. Children identified as malnourished were referred to the nearest health facility and information was provided to the respective municipalities about the number of malnourished children.

## Results

### Socio-Demographic Characteristics

Among the 300 mothers interviewed, majority (87.3%) were from the age group of 24–34 years and followed Hindu religion (84.7%). The proportion of Dalit participants among the ethnic group was the highest (32.7%). Richer group as per IWI (fourth quintile), consisted the majority (57%), followed by third quintile (18%). The majority children (70.3%) were aged above 24 months. More than half (53.7%) of the children were male (Table 1).

**Table 1:** Socio-demographic characteristics (n = 300).

	**Frequency (*n* = 300)**	**Percent (%)**
**Age of mother**	
<20 years	11	3.7
20–34 years	262	87.3
≥35 years	27	9.0
**Religion of mother**	
Hindu	254	84.6
Buddhist	32	10.7
Muslim	14	4.7
**Ethnicity of mother**	
Dalit	98	32.6
Disadvantaged indigenous ethnicity	37	12.3
Religious minorities	14	4.7
Relatively advantaged indigenous ethnicity	59	19.7
Brahmin and Chhetri	92	30.7
**Type of family**	
Nuclear	145	48.3
Joint	155	51.7
**Number of U5 children**	
Have only one child	267	89.0
Have two or more child	33	11.0
**The economic status of a family**	
Lowest quintile	20	6.7
Second quintile	12	4.0
Third quintile	54	18.0
Fourth quintile	171	57.0
Highest quintile	43	14.3
**Age of the child**	
<24 months	89	29.7
≥24	211	70.3
**Sex of the child**	
Male	161	53.7
Female	139	46.3

### Women Empowerment and Its Various Dimension

Around 60% of women had completed secondary level and above whereas few (3.3%) had never been to school. Almost every nine out of 10 women themselves took the decisions related to healthcare and household goods purchasing. More than 80% reported having freedom to visit their relatives whereas 62% were members of any community groups. More than one-third of women earned cash income independently and one-fourth were the owners of a house or land. In aggregate, nearly half of the mothers had a high empowerment level followed by moderate and low (Table 2).

**Table 2:** Maternal empowerment and its various dimensions.

**Empowerment level**	**Frequency (*n* = 300)**	**%**
Low empowerment	22	7.3
Moderate empowerment	131	43.7
High empowerment	147	49.0
Dimension of WEI^*^	
Decision making in healthcare	262	87.3
Decision making in household goods purchasing	260	86.7
Freedom to visit relatives	244	81.3
Having membership of community groups	186	62.0
Earn cash independently	110	36.7
Ownership of house/land	76	25.3
**Maternal educational status**	
No formal education	10	3.3
Primary education	111	37.0
Secondary and above education	179	59.7

* *WEI, Women's empowerment index*.

### Nutritional Status of the Children

Among 300 children, more than half (53.5%) were found to be stunted. The proportion of stunting among male and female children was almost similar at 25.5 and 27%, respectively. Similarly, severe and moderate stunting was noted among 5 and 21.7%, respectively. Around 14% were found wasted. Wasting among male child (9.4%) was almost two-fold as compared to female child (4.3%). Similarly, more than one third (34.7%) of the children were found underweight. The proportion of underweight among male children was slightly greater than the female child (19.2 vs. 15.8%, respectively). Overall, 60.3% children were felled under the normal category (with no stunting, wasting, and/or underweight) whereas remaining was categorized as having poor nutritional status (37.7%) (Table 3).

**Table 3:** Nutritional status of children, n (%).

	**Male (*n* = 161)**	**Female (*n* = 139)**	**Total (*n* = 300)**
**Stunting (height for age)**	
Severely stunted child	9 (5.6)	6 (4.3)	15 (5.0)
Moderately stunted child	32 (19.9)	33 (23.7)	65 (21.7)
Normal child	120 (74.5)	100 (72.0)	220 (73.3)
**Wasting (weight for height)**	
Severely wasted child	3 (1.9)	1 (0.7)	4 (1.3)
Moderately wasted child	12 (7.5)	5 (3.6)	17 (5.7)
Normal child	146 (90.6)	133 (95.7)	279 (93)
**Underweight (weight for age)**	
Severely underweight	5 (3.1)	2 (1.4)	7 (2.3)
Moderately underweight	26 (16.1)	20 (14.4)	46 (15.3)
Normal child	130 (80.8)	177 (84.2)	247 (82.3)
**Overall nutrition status**	
Poor nutritional status	63 (39.1)	56 (40.3)	119 (37.7)
Good nutritional status	98 (60.9)	83 (59.7)	181 (60.3)

### Association Between Women's Empowerment Level and Children's Nutrition Status

In preliminary analysis, only two background variables, maternal age (dummies; <26 or ≥26 yrs.) and number of children in a family (dummies; 1 or >1), were found significant with children's nutritional status, so entered as confounders in the multivariate models. Mothers with low empowerment were almost 30 (AOR = 29.5; 95% CI = 9.1–95.2) times; nearly 5 (AOR = 5.4; 95% CI = 1.1–27.1) times; nearly 29 (AOR = 29.4; 95% CI = 7.9–110.0) times; and 37 (AOR = 37.0; 95% CI =9.8–142.8) times more likely to have their children being stunted, wasted, underweight, and in poor (overall) nutritional status in reference to highly empowered mothers. Moderate empowerment level among women was also associated with children's stunting, underweight, wasting, and overall nutritional status but with lower strengths (Table 4).

**Table 4:** Strength of association between maternal empowerment and children's nutrition.

	**Child's nutritional status-frequency (%)**		**ξ^2^-statistic (*p*-value)**	**UOR (95% CI)**	**AOR (95% CI)^†^**	**Nagelkerke *R^**2**^***
	**Stunted**	**Normal**				
Low	14 (63.6)	8 (36.4)	74.05 (<0.001)^**^	35.0 (11.0–110.9)	29.5 (9.1–95.2)	0.368
Moderate	59 (45.0)	72 (55.0)		16.4 (7.1–37.7)	15.9 (6.9–36.8)	
High	7 (4.8)	140 (95.2)		Ref		
	**Wasted**	**Normal**				
Low	3 (13.6)	19 (86.4)	8.87 (0.012)^*^	5.6 (1.2–27.2)	5.4 (1.1–27.1)	0.074
Moderate	14 (10.7)	117 (89.3)		4.3 (1.4–13.4)	4.2 (1.4–13.3)	
High	4 (2.7)	143 (97.3)		Ref		
	**Under-wt**.	**Normal**				
Low	11 (50)	11 (50)	53.50^#^ (<0.001)^**^	35.8 (9.8–131.0)	29.4 (7.9–110.0)	0.294
Moderate	37 (28.2)	94 (71.8)		14.1 (4.9–40.8)	13.4 (4.6–39.1)	
High	4 (2.7)	143 (87.3)		Ref		
	**Malnourished**	**Normal**				
Low	7 (31.8)	15 (68.2)	31.53^#^ (<0.001)^**^	11.0 (3.3–36.9)	9.5 (2.7–32.7)	0.188
Moderate	33 (25.2)	98 (74.8)		7.9 (3.2–19.6)	7.6 (3.1–18.8)	
High	6 (4.1)	141 (95.9)		Ref		
	**Overall nutrition**					
	**Poor**	**Good**				
Low	19 (86.4)	3 (13.6)	90.86 (<0.001)^**^	42.7 (11.5–158.1)	37.0 (9.8–142.8)	0.399
Moderate	81 (61.8)	50 (38.2)		10.9 (6.0–19.8)	10.9 (5.9–20.0)	
High	19 (12.9)	128 (87.1)		Ref		

* *p < 0.05;*

** *p < 0.001;*

† *Adjusted to number of children and maternal age;*

# *Max. likelihood ratio*.

### Association Between the Various Dimension of Women Empowerment Level and Children's Nutritional Status

The results show that all the dimensions of the women's empowerment index were significantly associated with children's nutritional status. The decision making in health care, decision making in household good purchasing, having membership in the community, earning cash independently, ownership of house/land, and education status showed a strong association with children's nutritional status (Table 5).

**Table 5:** Association between various dimensions of maternal empowerment and children's nutrition.

**WEI dimension^**#**^**	**Nutritional status**, ***n*** **(%)**		**Df**	**ξ^2^**	***p*-value**
	**Good**	**Poor**			
**Decision making in health care**	
Yes	174 (66.4)	88 (33.6)	1	31.938	<0.001^**^
No	17 (18.4)	31 (81.6)			
**Decision making in household goods purchasing**	
Yes	173 (66.5)	87 (33.5)	1	10.674	<0.001^**^
No	8 (20.0)	32 (80.0)			
**Freedom to visit relatives**	
Yes	158 (64.8)	86 (35.2)	1	10.674	0.001^*^
No	23 (41.1)	33 (58.9)			
**Having membership of community group**	
Yes	131 (70.4)	55 (29.6)	1	20.850	<0.001^**^
No	50 (43.9)	64 (56.1)			
**Earning cash independently**	
Yes	97 (88.2)	13 (11.8)	1	56.283	<0.001^**^
No	84 (42.2)	106 (55.8)			
**Ownership of house/land**	
Yes	67 (88.2)	9 (11.8)	1	32.928	<0.001^**^
No	114 (50.9)	110 (49.1)			
**Education status**	
No formal education	2 (20.0)	8 (80.0)	2	19.879	<0.001^**^
Primary education	54 (48.6)	57 (51.4)			
Secondary education and above	125 (69.8)	54 (30.2)			

* *p < 0.05;*

** *p < 0.001*.

## Discussion

Children's nutritional status and the maternal empowerment level are analyzed and the findings are compared with that of the NDHS reports, other similar studies, and further discussed. Seminal literature has confirmed strong relationship between early childhood undernourishment and lowest income groups (26). This causality has been documented in diverse world regions ranging from Sub-Saharan Africa (27), Syria (28), India (15, 29), Yugoslavia (30), and both Koreas (31) alike. This study showed 26.7, 7, and 17.6% of children being stunted, wasted, and underweight, respectively. These are less than the findings of the NDHS preliminary report 2016 (21) at national level, but, are very close to the data of Gandaki province where the study areas (rural municipalities) are located, i.e., 28.9% stunting, 5.8% wasting, and 14.9% underweight. The contrasting figures of the study findings with that of national level may be due to higher socio-economic and literacy rates of Kaski district.

The study showed that low and moderate level of empowerment (with reference to high) were moderate to strongly associated with undernutrition of the children: Stunting (AOR, around 30 and 16, respectively), wasting (AOR, 5 and 4, respectively), and underweight (AOR, 29 and 13, respectively) (all *p* < 0.05). These findings are consistent with another study in Nepal, which found women's empowerment positively associated with nutritional status of their children (32). Nonetheless the study from Pakistan showed an insignificant effect on child nutrition (33). However, in a systematic review of 39 studies, only 20% of all the significant associations (weighted) showed relationship of higher women empowerment with lower children's nutritional status, and 3% (weighted) were in the opposite direction (13).

This study showed that women's involvement in household decision-making (a component of empowerment) was statistically significant with the nutritional status of children. These verdicts were accord with another study from Northern Benin, which showed positive correlation between women's empowerment and leadership while decision making was correlated with wasting and underweight among children (34). An RCT conducted in Burkina Faso also showed that improved women empowerment, especially in decision making in health and household purchasing, contributed to reducing the wasting of their children (16). These findings also harmonize with ours.

Our study showed that women's engagement in a community group was associated with child nutrition. The result is coinciding with the cross-sectional study from Andhra Pradesh, which showed that larger and more literate social networks are associated with better length-for-age of 1-year-old children (35). Another study from India found that high maternal cognitive social capital was associated with the highest level of stunting (36). Children in communities with a high proportion of women autonomy in healthcare or movement or money separately had a lower risk of being stunted, underweight, or wasted (37).

In this study, the cash earning that leads to higher women's empowerment level was strongly associated with child nutrition. The finding concurred with the study from rural Karnataka, which showed a strong association with maternal employment and children's underweight (38).

In this study, although ownership of house or land that comprised a part of women's empowerment, was associated with children's nutrition, the findings are consistent with the study from Nepal which found that women who own land are significantly more likely to have the final say in household decisions (OR = 1.48), a measure of empowerment (37) but a study from Uganda found that ownership exhibited no differentials with child stunting (39). When only land/house ownership should be taken, it should be reassessed with further clarifications.

In this study, the level of education, which is a factor for high women's empowerment, was strongly associated with a child's nutrition. The finding is accordant with the study from Nairobi that found mother who passed the primary level have high (43%) stunted children compared to the mothers who passed the secondary level of education (40). Our study is also unfailing with the study from the region of Tanzania which showed that maternal education is one of the predictors of stunting (OR 2.31; 95% CI: 1.43–3.64) and parental education as the predictor of underweight (OR 1.76; 95% CI: 1.07–2.89) (41). The children whose parents are illiterate or having a low level of education are more vulnerable to the nutritional problem. These findings coincide with several previous studies (42–46). Similarly the another study from a country of east Africa, Mozambique which used Multiple Indicators Multiple Causes (MIMIC) shows the educational level of the mother are positively correlated with the nutritional status of children (17). So, parental education is, probably, the most consistent factor found associated with child's health and nutritional outcome. Furthermore, the maternal education has shown to be an essential factor driving female inclusion into the workforce of domestic society, increasing their income and social security (47). Degree of sexual revolution taking place in an observed society is also closely related to the emancipation of women and their absorption into the labor market (48). This was proven to be a lengthy historical process with way different outcomes and stages in different world regions from Latin America (49) to the South Asia (50) and ASEAN (51). Final consequences turned out to be mostly positive in terms of growing female wages and social independence capacity inclusive of ability to raise children (52). Yet male—female disparity in revenues remains significant even amongst some of the wealthiest OECD countries (53). In addition, alternative social protection financial mechanisms were created in exceptionally rich societies such as Switzerland (54) or Japan to provide financial support to unemployed mothers raising their own children in the capacity of housewives (55). Such solutions remain hardly accessible to less wealthy economies (56).

However, it is not clear from the literature that how women's empowerment is associated with children's nutritional status. A systematic review summed up (from 62 quantitative studies) as 82 and 84 percent of the weighted cases, stunting and wasting, respectively, were not found significant with women empowerment. For this, the authors have blamed the study designs rather than the embedded underlying associations (13).

To sum up, education (40, 41), income (47), and social protection for the unemployed (53, 55), decision making (16), sexual and reproductive health rights (48–51), and social networking and engagement (35, 36) have been discussed as the sustainable and long-term strategies. In addition, nutritional gain through behavior change communication focusing on knowledge and skills, increased control over income from the sale of targeted commodities (57), family approach with fixed empowerment goal, and problem reflection and critical thinking (58) can be taken as instantaneous strategies which are significant for nutrition-sensitive interventions, too. Although there is a variation of indicators of women's empowerment scale the internal agency component, which includes spousal communication, attitude toward intimate partner violence, seems promising and so, be taken for granted and applied in policy cautiously. Financial autonomy further needs to be tested with trial study whereas decision making with longitudinal design. Developing the context-specific valid scale is also deemed imperative.

## Conclusion

Our results demonstrate that 7%, more than one in every four, and more than one in every six children were wasted, stunted and underweight, respectively. Similarly, almost half of the mothers were highly empowered. There was nearly a five-fold increase in odds of wasting, thirty-fold increase in odds of stunting and twenty-nine-fold increase in underweight, among children whose mother had low empowerment status compared to their counterparts. Empowerment dimensions like women's education and their community membership are cautiously warranted so as to impact on their decision making, thereby positively contributing to children's nutritional status. Dimensions of material resources like independent cash earning and house/land ownership need further empirical evidences from stronger study designs.

## Data Availability Statement

The raw data supporting the conclusions of this article will be made available by the authors, without undue reservation.

## Ethics Statement

The studies involving human participants were reviewed and approved by Pokhara University Research Center (PURC), Pokhara University, Nepal. The patients/participants provided their written informed consent to participate in this study.

## Author Contributions

SP and CA conceptualized the research and analyzed the data. SP collected the data. SP, CA, RKY, and MJ prepared the first draft. SP, CA, DKY, and DKT prepared the second draft by addressing the comments from the reviewers. All authors reviewed the first draft, contributed for improvement, reviewed the final draft, read, and agreed to the final version of the manuscript.

## Conflict of Interest

The authors declare that the research was conducted in the absence of any commercial or financial relationships that could be construed as a potential conflict of interest.

## Publisher's Note

All claims expressed in this article are solely those of the authors and do not necessarily represent those of their affiliated organizations, or those of the publisher, the editors and the reviewers. Any product that may be evaluated in this article, or claim that may be made by its manufacturer, is not guaranteed or endorsed by the publisher.
